# Predicting 30-Day Readmissions: Performance of the LACE Index Compared with a Regression Model among General Medicine Patients in Singapore

**DOI:** 10.1155/2015/169870

**Published:** 2015-11-23

**Authors:** Lian Leng Low, Kheng Hock Lee, Marcus Eng Hock Ong, Sijia Wang, Shu Yun Tan, Julian Thumboo, Nan Liu

**Affiliations:** ^1^Department of Family Medicine & Continuing Care, Singapore General Hospital, Academia Level 4, 20 College Road, Singapore 169856; ^2^Department of Emergency Medicine, Singapore General Hospital, Outram Road, Singapore 169608; ^3^Health Services and Systems Research, Duke-NUS Graduate Medical School, Singapore; ^4^Integrated Health Information Systems, Singapore; ^5^Department of Rheumatology & Immunology, Singapore General Hospital, Singapore; ^6^Centre for Quantitative Medicine, Duke-NUS Graduate Medical School, Singapore

## Abstract

The LACE index (length of stay, acuity of admission, Charlson comorbidity index, CCI, and number of emergency department visits in preceding 6 months) derived in Canada is simple and may have clinical utility in Singapore to predict readmission risk. We compared the performance of the LACE index with a derived model in identifying 30-day readmissions from a population of general medicine patients in Singapore. Additional variables include patient demographics, comorbidities, clinical and laboratory variables during the index admission, and prior healthcare utilization in the preceding year. 5,862 patients were analysed and 572 patients (9.8%) were readmitted in the 30 days following discharge. Age, CCI, count of surgical procedures during index admission, white cell count, serum albumin, and number of emergency department visits in previous 6 months were significantly associated with 30-day readmission risk. The final logistic regression model had fair discriminative ability* c*-statistic of 0.650 while the LACE index achieved* c*-statistic of 0.628 in predicting 30-day readmissions. Our derived model has the advantage of being available early in the admission to identify patients at high risk of readmission for interventions. Additional factors predicting readmission risk and machine learning techniques should be considered to improve model performance.

## 1. Introduction

Patients at high risk of readmission are a strain to healthcare system and contribute to bed shortage. Healthcare resources are finite and such frequent readmissions can overwhelm even developed health systems. Patients with frequent admissions also experience significant psychological stress and financial burden. In the United States, 30-day readmissions are considered an accountability measure and quality indicator, and the Centers for Medicare and Medicaid Services (CMS) adjusts reimbursements to hospitals according to readmission rates.

Reducing readmissions is a priority of the Ministry of Health (MOH), Singapore. Singapore's population is one of the most rapidly ageing in Asia and faces the challenge of healthcare demand exceeding supply. An estimated one million or 20% of the country's population will be elderly by 2030 [1]. The all-cause 30-day readmission rate in Singapore in 2010 was 11.6% [2], rising to 19.0% in those aged 65 and older. This rate is only slightly lower than in the United States [3]. The gap between the lesser developed community care and the high specialized acute care services is widely acknowledged. Transitional care programs are started by acute care hospitals to ensure a safe transition of patients from hospital to home. Currently, the planning for transitional care interventions by various hospitals is challenging as there is a lack of understanding on the prevalence of patients at various risk strata for readmission. Without accurate stratification, there could be under- or overdevelopment of the relevant transitional care programs that are meant to target patients of a certain risk stratum.

The reasons behind readmission are many and can range from unresolved medical issues and poor transition of care to unanticipated new problem in the postdischarge period. Interventions to mitigate this readmission risk are resource intensive and should be targeted at patients at high risk of readmission. A model to predict patients at risk of readmission will help healthcare administrators and providers to allocate appropriate resources to patients at highest risk of readmission.

Several countries have developed predictive models for readmission, for example, LACE and LACE+ index [4, 5] in Canada, PARR-30 [6] by the UK National Health System, and the HOSPITAL score in the United States [7]. Many of these models have limited generalizability to other health systems due to the unique variables derived from their respective specific settings [6, 8, 9]. The LACE index (length of stay, acuity of admission, Charlson comorbidity index, and emergency department visits in past 6 months), derived in Ontario, Canada (Table 1), is simple to use and may have potential clinical utility in Singapore. However, the LACE index was developed from middle-aged Canadian patients free of serious comorbidities, and its applicability to another population needs to be validated before clinical utility. Tan et al. [10] found that medical patients in Singapore with a LACE score ≥ 10 had an almost 5 times higher risk of 30-day unplanned readmission after index discharge. But the LACE index can only be calculated on discharge and performance of the LACE index was not validated in our population. A simple predictive model using variables available early in the admission will identify patients to receive intensive interventions to reduce their readmission risk.

Our primary objective is to validate the LACE index and compare its performance with a derived regression model to predict 30-day readmission risk among general medicine patients in Singapore.

## 2. Materials and Methods

### 2.1. Setting and Study Population

Singapore is a multiethnic Asian country with a population of 5.6 million. The public healthcare system is divided into six regional health systems, each responsible for a specific geographical region. Singapore General Hospital (SGH) is the flagship hospital of the SingHealth regional health system and the largest tertiary hospital in Singapore with 37 clinical specialities and 88,000 inpatient admissions each year [11].

All adult patients ≥21 years, with alive-discharge episodes from the Department of Internal Medicine, SGH, in 2012 were eligible for inclusion. Internal Medicine is the general medical service and the largest medical speciality in SGH that discharges about 18% of all hospitalized patients per year. Patients who died during the index admission, who were nonresidents, or who had a discharge destination other than home at index discharge were excluded from analysis. The first admission in 2012 is the index admission and we counted no more than one readmission for each internal medicine patient discharged in 2012.

### 2.2. Data Collection, Variables, and Outcome Measures

We extracted deidentified data from the electronic health intelligence system (eHINTS) of SGH using the Oracle Business Intelligence and Enterprise Edition (OBIEE) Software. The eHINTS consolidates and analyses patient and healthcare data that are uploaded on the web-based business intelligence software. Variables were chosen a priori and according to medical literature. These variables are capable of extraction from the hospital's electronic health records (EHR) and available to all hospitals in Singapore, with potential for external validation of our model. The comorbidities were identified using International Classification of Diseases (ICD-10) codes in any primary or secondary diagnosis fields dating back to one year preceding the index admission. The Charlson comorbidity index (CCI) was computed for each patient.

In addition to the four variables in the LACE index, variables extracted included patient demographics (age, gender, marital status, race, and admission ward class as a marker of socioeconomic status), comorbidities (major diseases listed under the Charlson comorbidity index, number of medications on discharge), clinical and laboratory variables during the index admission (haemoglobin, white cell count, c-reactive protein, serum sodium, serum creatinine, serum albumin, and number of surgical procedures), and prior healthcare utilization in the preceding year (number of admissions, number of emergency department visits, and number of specialist outpatient clinic visits).

### 2.3. Statistical Analysis

The demographics and baseline characteristics of the study population were described and compared among patients with and without meeting the outcome (30-day readmission). Continuous variables were presented as means (standard deviation) by using Student's *t*-test in the analysis or medians (interquartile range) by using Wilcoxon rank-sum test. Categorical variables were analysed with chi-square test or Fisher's exact test when appropriate and were presented as numbers (percentage). Statistical significance was set as *P* < 0.05.

Significant variables (e.g., age, length of stay, Charlson comorbidity index, LACE score, emergency department visits in previous 6 months, admission ward class, and laboratory variables) selected from univariate analysis were associated with 30-day readmissions using multivariate logistic regression where stepwise forward variable selection was implemented to build the prediction model. In the final regression model, we only included variables that would be available early in the admission for the model to be useful to clinicians to identify their patients at high risk of readmission. The receiver operating characteristic (ROC) analysis was conducted to evaluate the predictive performance for both LACE index and the regression model, and* c*-statistic (i.e., area under the ROC curve) was reported. Data analyses were conducted in IBM SPSS Statistics 21 (IBM Corporation, Armonk, NY) and MATLAB R2014b (Mathworks, Natick, MA).

## 3. Results

**Figure 1:**
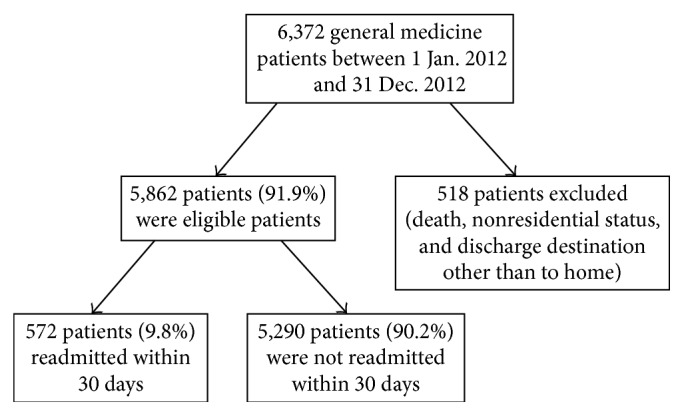
Study flow chart showing number of included, excluded, and readmitted patients.

6,377 unique patients were admitted to the internal medicine wards in 2012. Of these, 515 (8.1%) were excluded from analysis because of death during the index admission, nonresidential status, or having a discharge destination other than home at index discharge (Figure 1).

Of the 5,862 (91.9%) patients remaining in the cohort, 572 patients (9.8%) were readmitted in the 30 days following discharge (Figure 1). Patient characteristics are shown in Table 2. The mean age of all patients was 60.0 (SD = 19.5) years. The majority were female (55.3%, *n* = 3,241). Most admissions were emergent (99.4%, *n* = 5,792). Median length of stay was 3 days (IQR 2–5). On average, patients had a median LACE score of 6 (IQR 5–8). The readmitted patients were older compared to the nonreadmitted patients and had significantly longer length of stay during the index admission, higher CCI, more ED admissions in the preceding 6 months, and higher LACE scores (Table 2).

**Figure 2:**
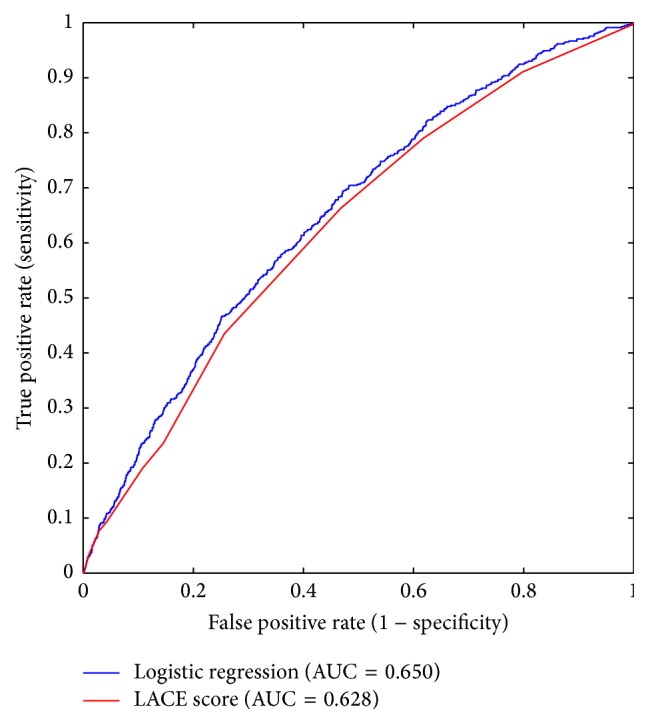
Comparison of the receiver operating characteristic (ROC) curves for the LACE score and the derived logistic regression model.

In a multivariate logistic regression, six variables were found to be significantly associated with readmission within 30 days of discharge (Table 3). Five were included in the final model: age, CCI, white cell count, serum albumin, and emergency department visits in previous 6 months. We excluded count of surgical procedures during index admission as this information is not available early in the admission to be clinically useful. The final logistic regression model had fair discriminative ability (*c*-statistic of 0.650, 95% CI: 0.625–0.676) (Figure 2). As a comparison, the LACE index achieved a* c*-statistic of 0.628 (95% CI: 0.602–0.653) in predicting 30-day readmissions. The optimal cut-off for the LACE index is a score of 6 or more with a sensitivity of 66.3% and specificity of 53.3%. The optimal cut-off for the regression model has comparable sensitivity and specificity of 61.4% and 60.3%, respectively (Table 4).

## 4. Discussion

We were able to identify key patient-level predictors of readmission and derive and internally validate a model for assessing readmission risk in general medicine patients hospitalized for a variety of medical conditions and discharged home. Several patient-level factors identified as significant predictors were known from the published literature, such as age, the Charlson comorbidity index [4], and number of emergency department visits in the preceding 6 months. The number of surgical procedures, white cell count, and serum albumin levels are clinical markers of patients' condition during the index admission and the frailty of the patients [12], respectively. In contrast to the LACE index, length of stay and acuity of admission were not associated with risk of readmission within 30 days after adjusting for covariates in the multivariate logistic regression model. It is possible that, in our cohort, the duration of admission was affected by other factors such as social circumstances and did not reflect the severity of illness entirely. Almost all admissions were emergent and reduced its predictive ability for readmission risk.

The discriminative ability of our model is only fair but comparable to the discriminative ability of other commonly cited readmission risk prediction models [4, 6, 13, 14]. It is likely that important predictors of readmission such as level of social support, function, and financial payment modes, for example, insurance, government welfare, or self-payment, affect patients' healthcare utilization patterns [15] but are not available from our administrative database. A model that incorporated these variables with eight interaction terms had a* c*-statistic of 0.83 but may be difficult to deploy clinically due to its complexity and possible overfitting for the population used to derive it [16].

When we applied the LACE index to our cohort of general medicine patients, we found that the LACE index had poorer discriminative ability (*c*-statistic 0.628), compared to our derived model (*c*-statistic of 0.650) and the Canadian population from which it was derived (*c*-statistic of 0.69). This poorer performance in our cohort is consistent in other populations that validated the LACE index [17]. The difference in performance is not surprising, as the characteristics of the cohort enrolled in the Canadian study were of a lower risk profile and differed from those of the usual patients who attend general medicine wards in Singapore. Patients in the Canadian cohort were mainly free of serious comorbidities, while our general medicine cohort had higher CCI scores, more medications dispensed on discharge, and greater utilization of hospital services preceding the index admission. When we evaluated the discriminative ability of the LACE index in our cohort stratified by age, the performance of LACE in older patients ≥ 65 years dipped to* c*-statistic 0.59. This is consistent with the performance of LACE in an older UK and Denmark population [17, 18].

The fair discriminative ability of both the LACE index and our derived model suggests much room for improvement to the model. Future models should consider more clinical, social, and functional variables for inclusion but need to be balanced against the costs of collecting these additional risk factors prospectively. The SingHealth regional health system has developed its enterprise analytic platform featuring a single enterprise data repository that integrates information from multiple healthcare transactional systems including applications from administration and clinical and ancillary services [19]. The quality information is hosted on user-friendly web-based Business Intelligence/Dashboard front-end software, and can be easily accessed online by researchers and healthcare providers. Analytical tools with “self-service” drill down capabilities enable faster and more efficient analysis. With the expansion of the data repository in our health system and health systems worldwide, advanced machine learning techniques will be needed to handle the data and to improve the performance of prediction models for various health outcomes [20, 21] and should be considered as an alternative method for model derivation.

### 4.1. Limitations

Our study has a number of limitations. Firstly, our prediction model was derived from administrative data, which may have coding errors. Secondly, as a retrospective study, our database is limited to variables routinely collected into the EHR. Other factors that may contribute to readmission risk such as the level of social support, functional limitations, and frailty of patients are not included in this study. Thirdly, we did not adjudicate whether each readmission was elective versus unplanned. However, based on our collective experience, we would expect the rate of elective readmissions on general medicine services to be low. Fourthly, we were unable to confirm out of hospital deaths or readmissions to hospitals other than ours, although only a minority utilized services from more than one hospital and majority of elderly die in hospitals in Singapore [22, 23]. Finally, although our study was conducted at the largest hospital in Singapore, caution should be exercised in generalizing its findings to other medical departments or other hospitals without externally validating the model.

## 5. Conclusions

Our derived model had comparable discriminative ability compared to the LACE index in our cohort of general medicine patients in Singapore. Our model has the advantage of being available early in the admission to identify patients at high risk of readmission to receive interventions to prevent potentially avoidable readmissions. Additional factors that predict readmission risk and advanced machine learning techniques should be considered to improve readmission models. The developing enterprise analytic platform in our health system will further build capabilities in predictive analytics in Singapore.

## Figures and Tables

**Table 1:** Components of the LACE index.

Variable	Value	Points
Length of stay, days	<1	0
	1	1
	2	2
	3	3
	4–6	4
	7–13	5
	≥14	7

Acute (emergent) admission	Yes	3

Charlson comorbidity index score	0	0
	1	1
	2	2
	3	3
	≥4	5

Emergency department visits during previous 6 months	0	0
	1	1
	2	2
	3	3
	≥4	4

The LACE score is calculated by summing the points of the above 4 variables.

**Table 2:** Patient characteristics and their association with readmission within 30 days.

	All patients (*n* = 5862)	Readmitted patients (*n* = 572)	Nonreadmitted patients (*n* = 5290)	*P* value
Age, mean (SD)	60.0 (19.5)	66.6 (17.0)	59.3 (19.6)	<0.001
Male (%)	2621 (44.7%)	264 (46.2%)	2357 (44.6%)	0.479
Length of stay, median (IQR)	3 (2–5)	4 (2–8)	3 (2–5)	<0.001
Urgent admission (%)	5827 (99.4%)	568 (99.3%)	5259 (99.4%)	0.772
Charlson comorbidity index score, mean (SD)	0.256 (0.615)	0.385 (0.755)	0.242 (0.597)	<0.001
LACE score, median (IQR)	6 (5–8)	7 (6–8)	6 (5–8)	<0.001
ED visits in previous 6 months, mean (SD)	0.132 (0.438)	0.154 (0.415)	0.129 (0.440)	0.033
Admission ward class B2/C (%)	4799 (81.9%)	495 (86.5%)	4304 (81.4%)	0.002
Number of surgical procedures, mean (SD)	0.170 (0.573)	0.308 (0.828)	0.155 (0.536)	<0.001
Number of dispensed medications at discharge, mean (SD)	8.753 (7.995)	10.920 (8.075)	8.519 (7.952)	<0.001
Congestive cardiac failure (%)	185 (3.2%)	33 (5.8%)	152 (2.9%)	0.001
Cerebrovascular disease (%)	34 (0.6%)	1 (0.2%)	33 (0.6%)	0.250
Chronic obstructive pulmonary disease (%)	47 (0.8%)	6 (1.0%)	41 (0.8%)	0.457
Any malignancy (%)	8 (0.1%)	5 (0.9%)	3 (0.1%)	<0.001

SD: standard deviation; IQR, interquartile range; ED: emergency department.

**Table 3:** Variables in the final logistic regression model for prediction of 30-day readmissions.

Variable	Beta coefficient	Adjusted odds ratio (95% CI)	*P* value
Age	0.018	1.019 (1.013–1.024)	<0.001
Charlson comorbidity index	0.213	1.238 (1.097–1.396)	0.001
White cell count	0.038	1.039 (1.003–1.077)	0.035
Serum albumin	−0.057	0.944 (0.927–0.962)	<0.001
ED visits in previous 6 months	0.239	1.270 (1.062–1.519)	0.009

CI: confidence interval; ED: emergency department.

**Table 4:** Sensitivity and specificity for the regression model and LACE indices greater than 10 and 6.

Model	Readmitted (*n* = 572)	Not readmitted (*n* = 5290)	Sensitivity	Specificity
LACE index with cut-off of 10				
Lower risk	519	5068	9.3%	95.8%
Higher risk	53	222		
LACE index with optimal cut-off of 6				
Lower risk	193	2820	66.3%	53.3%
Higher risk	379	2470		
Regression model using variables available early in the admission *with optimal cut-off*				
Lower risk	221	3190	61.4%	60.3%
Higher risk	351	2100		
